# The efficacy of recombinant versus urinary HCG in ART outcome

**Published:** 2012-11

**Authors:** Maryam Eftekhar, Mohammad Ali Khalili, Elham Rahmani

**Affiliations:** 1 *Department of Obstetrics and Gynecology, Research and Clinical Center for Infertility, Shahid Sadoughi University of Medical Sciences, Yazd, Iran.*; 2 *Department of Embryology, Research and Clinical Center for Infertility, Shahid Sadoughi University of Medical Sciences, Yazd, Iran.*; 3 *Department of Obstetrics and Gynecology, Bushehr University of Medical Sciences, Bushehr, Iran.*

**Keywords:** *Recombinant HCG*, *Urinary HCG*, *Assisted reproduction technology*, *Ovulation*, *Final follicular maturation*

## Abstract

**Background:** Human chorionic gonadotropin (HCG) has been used as a replacement for the mid-cycle luteinizing hormone (LH) surge for several years. The recent arrival of recombinant DNA technology has made recombinant HCG (rHCG) accessible.

**Objective:** To assess efficacy of rHCG compared to urinary HCG (uHCG) for triggering of ovulation and induction of final oocyte maturation in assisted reproductive cycles.

**Materials and Methods:** 200 patients who were candidate for ICSI were randomly divided in two groups. In group I (rHCG), patients received 250µg of rHCG for final oocyte maturation, and in group II (uHCG) the patients received 10000 IU of uHCG. Measured outcomes were number of retrieved oocyte and mature oocyte, maturation rate of oocyte, fertilization rate and clinical pregnancy rate.

**Results:** The rates of oocyte maturity were similar in both groups. Fertilization rate was similar in two groups (58.58% in rHCG group versus 60.58% in uHCG group p=0.666). The clinical pregnancy rate per cycle was similar in both group 34.0% in rHCG group versus 39% in uHCG group (p=0.310).

**Conclusion:** We demonstrated that rHCG is as effective as uHCG, when it is used for final oocyte maturation in ICSI cycles. The numbers of retrieved oocyte and maturation rates were similar in both groups; also fertilization and clinical pregnancy rates were similar.

## Introduction

The preovulatory surge of luteinizing hormone (LH) performs physiological stimulation for final oocyte maturation and causes ovulation (1-4). After LH surges the process of meiosis progress, the oocyte completes meiosis I and starts meiosis II, the oocyte and cumulus cells separates from the follicle wall and eventually lead to release of the oocyte-cumulus complex (5).

The mature oocyte is then picked up by fimbrial end of fallopian tube (1). Throughout assisted reproduction cycles, premature LH surge affects cycle outcomes. Premature LH surge could lead to premature ovulation and as a result, interfere with oocyte collection during cycles. Premature luteinization also negatively affects egg quality and synchronization between embryos and endometrium. For prevention of LH surge GnRH agonist or antagonist is used in combination with gonadotropin in ART cycles (6, 7). 

Therefore, LH surge is needed to create by consumption of exogenous hormones for final oocyte maturation and ovulation induction in ART (8). Human chorionic gonadotropin (HCG) is produced by the trophoblasts from the early days of implantation which supports the corpus luteum and feto-placental communication (9-11). 

HCG is a glycoprotein and structurally is similar to LH; both hormones have similar effect and bind to the same receptors. UHCG is collected from urine of pregnant women, which has been clinically used for triggering of ovulation and luteinization in anovulatory women (12). For at least 3 recent decades, uHCG has also been used for final oocyte maturation and luteal phase support (11, 13, 14). 

However, consumption of UHCG is associated with a number of disadvantages, the source of drug is urine of pregnant women and it is an uncontrolled source. Therefore purity of uHCG could differ from batch-to-batch and eventually lead to variation in activity and result (15).

On the other hand, rHCG is derived from genetically engineered Chinese hamster ovary cells by recombinant DNA technology. The high purity of this product makes the drug suitable for subcutaneous injection, and is self-injection (16). The aim of study was to assess the efficacy of rHCG compared to uHCG for triggering the ovulation and induction of final oocyte maturation in assisted reproductive cycles. 

## Materials and methods

This study was conducted at Yazd Research and Clinical Center for Infertility, affiliated to Shahid Sadoughi University of Medical Sciences from January 2009 to July 2010. The study was approved by Ethic Committee, and written informed consent was taken from all participants. 200 Women with regular menstrual cycles, age <38 years old, FSH <10IU/L and BMI <30kg/m^2^ were included in this study. 

**Figure 1:**
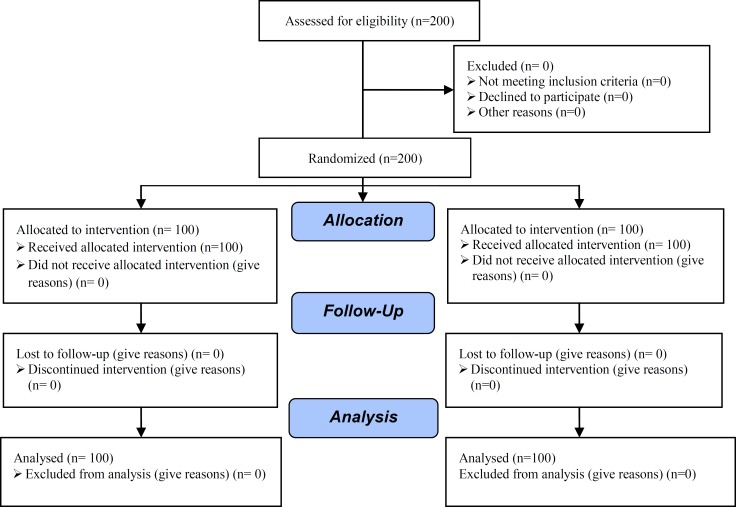
Consort flow chart of RCT.

The exclusion criteria was history of metabolic or endocrine disorder, history of pelvic surgery and severe male factor infertility which was defined as severe oligospermia (<5million sperm/ml), asthenospermia (5% progressive motility), or teratospermia (4% normal forms by strict criteria). The study was a prospective randomized study and allocation of the patients into two groups was done alongside by using packets which included computerized randomization (figure 1). Pituitary desensitization was done for both groups by administration of Decapeptyl (Decapeptyl® 0.1 mg, Ferring, Germany) 0.1 mg per day subcutaneously from previous mid-luteal phase, and was continued until the day of HCG injection. Pituitary desensitization confirmed by sonography in second day of cycle. 

If endometrial thickness was less than 5 mm in diameter and ovarian cyst more than 10 mm in diameter was not seen, then ovarian stimulation was started from second day of menstrual cycle by administration of (150 IU) of human recombinant follicle-stimulating Hormone (Gonal-F, Serono, Aubnne, Switzerland). It was injected subcutaneously and dose of Gonal-F was adjusted according to patients’ response then continued until the day of HCG injection. Monitoring of cycle was done by serial vaginal ultrasonography and measurement of serum estradiol level. 

When at least two dominant follicles reached to 18mm in mean diameter or two follicles with mean diameter larger than 16 mm diameter and one by mean diameter >18 mm was observed in vaginal sonography, the patients were divided into two groups. In group I (rHCG), 100 patients received a single subcutaneous injection of 250 µg rHCG (Ovidrel). In group II (uHCG), 100 patients received a single intramuscular injection of 10000 IU urinary HCG (pregnyl, ® organon, oss, Netherlands). In both group oocyte retrieval was done 34-36 hours after HCG injection by using a 17 gauge needle under ultrasound guidance through the vaginal rout. 

The numbers of retrieved oocyte were recorded. Approximately two hours after retrieval cumulus and corona radiate cell were removed gently under an inverted microscope. Metaphase II oocytes (mature oocytes) were characterized by the presence of first polar body, metaphase I oocytes were characterized by absence of both germinal vesicle and first polar body and prophase I oocytes were characterized by its distinct germinal vesicle. Intra cytoplasmic injection (ICSI) was done for all cycles and only mature oocytes were injected. 

About 18 hours after microinjection, oocytes were evaluated for fertilization presence of pronuclear and two polar bodies were considered as normal fertilized oocyte. Embryos were transferred 2 days after oocyte retrieval. Good quality embryos were transferred in both groups by using Labotect catheter (Labtect, Gottingen, Germany). Luteal phase support was done by intra muscular injection of 100 mg progesterone (progesterone, Aburaihan CO, Tehran, Iran), starting from day of oocyte retrieval and continued until observation of fetal heart activity by vaginal sonography. 

If estradiol level reached greater than 3000pg/ml and /or more than 15 follicles with greater than 14 mm in diameter was observed in each ovary by vaginal sonography, the patient was considered at risk of ovarian hyper stimulation syndrome (OHSS). The primary outcome was the numbers of mature oocyte, maturation rates of oocyte was defined as the numbers of mature oocyte retrieved per numbers of retrieved oocyte. Chemical pregnancy was defined as serum βHCG >50IU/L measured 2 weeks after embryo transfer. Clinical pregnancy was defined as observation of fetal heart activity by ultrasonography 3 weeks after positive βHCG. 

**Statistical Analysis**

Statistical analysis was carried out using the statistical package for the social science (SPSS version 15.0 for windows, Chicago, IL). Both t-test and chi-square test were used to detect significant differences (p<0.05) of the all variables between the two groups.

## Results

Basic characteristics of patients regarding female age, duration of infertility, basal FSH and etiology of infertility was statistically similar in both groups (Table I). The cycle’s characteristics and ART outcomes is presented in table II. Duration of stimulation, dose of FSH (IU) used, numbers of follicle >14 mm in diameter, numbers of retrieved oocyte and mature oocyte, maturation rate of oocyte and numbers of embryos transferred were statistically similar in both groups. 

Fertilization rate was similar in two groups (58.58% in rhCG group versus 60.58% in uhCG group; p=0.666). Clinical pregnancy rate per cycle was similar in both groups (34.0% in rhCG group versus 39% in uhCG group; p=0.310). Embryo transfer was cancelled in 7 cycles in rHCG group and 8 cycles in uHCG group due to risk of mild to moderate OHSS (p=1.000). No case of severe OHSS was seen. However none of these patients excluded from the final analysis.

**Table I:** Basic characteristic of patients undergoing ICSI

**Variable**		**rHCG (group I) (n=100)**	**uHCG (group II) (n=100)**	**p-value**
Age(year)		29.63 ± 4.85	29.30 ± 4.89	0.637
Duration of infertility (years)		9.01 ± 6.66	8.98 ± 7.45	0.988
Basal FSH(IUI/L)		7.65 ± 3.62	6.75 ± 3.35	0.125
Infertility causes				0.382
	Male factor (%)	41 (41)	43 (43)	
	Tubal factor (%)	6 (6)	11 (11)	
	Unexplained factor (%)	25 (25)	20 (20)	
	Mild endometriosis (%)	4 (4)	4 (4)	
	Mixed (%)	13 (13)	6 (6)	
	Ovarian factor (%)	10 (10)	16 (16)	

Parameters expressed as mean±SD or percentages, as appropriate.

**Table II:** Cycle characteristic and ICSI outcome

**Variable**	**rHCG (group I) (n=100)**	**uHCG (group II) (n=100)**	**p-value**
Duration of stimulation(d)	11.24 ± 2.26	11.33 ± 1.90	0.753
Dose of FSH used (IU)	2028 ± 680	2154 ± 894	0.267
No of follicle >14 mm	12.34 ± 12.5	11.81 ± 671	0.716
No. of retrieved oocytes	9.75 ± 6.65	9.64 ± 7.29	0.920
No. of mature oocytes	8.34 ± 5.27	8.27 ± 6.44	0.935
Maturation rate of oocytes	87.99%	86.25%	0.543
No. of transferred embryos	2.30 ± 0.90	2.09 ± 1.15	0.194
Fertilization rates	58.58%	60.58%	0.666
Clinical pregnancy rates	34%	39%	0.310
Abortion (%)	4 (11.76%)	5 (12.8%)	1.000
Mild to moderate OHSS (%)	7 (7%)	8 (8%)	1.000
Endometrial thickness	9.49 ± 1.63	9.82 ± 1.59	0.221

Parameters expressed as mean ± SD or percentages, as appropriate.

## Discussion

Our study was done for evaluation of efficacy of rHCG compared to uHCG. According to our findings; we demonstrated that rHCG is as effective as uHCG, when it is used for final oocyte maturation in ICSI cycles. The numbers of retrieved oocyte and maturation rates were similar in both groups; also fertilization and clinical pregnancy rates were similar. In contrast to our study, Geneva compared rHCG and uHCG, and demonstrated that despite administration of similar doses, rHCG was associated with higher numbers of mature oocytes per cycles. He suggested that HCG degenerated products in the urinary preparation have slight interference with the active HCG molecules and consequently with the HCG induced oocyte maturation (3, 11). 

In consistent with Geneva, Farag *et al* analyzed the effect of rHCG on oocyte nuclear and cytoplasmic maturity compared to uHCG when it used for inducing ovulation. They showed that rHCG resulted in a statistically higher rates of mature oocytes regarding nuclear and cytoplasmic maturity (1). Similar to our study, Driscoll *et al* in a prospective and randomized study compared rHCG and uHCG for inducing oocyte maturation and follicular luteinization in ovarian stimulation. Primary outcome including number of retrieved oocytes per follicles, the number of mature oocytes and fertilization rate were similar in both groups. 

On the other hand, they showed that progesterone concentration 6-7 days after HCG administration was greater in the rHCG group than in uhCG group. Therefore they suggested that whether this would allow a more favorable luteal environment for implantation will require further investigation (17). Meike *et al* also compared clinical outcomes of rHCG and uHCG in fresh non donor cycles. They showed that rate of mature oocytes, fertilization and clinical pregnancy were similar in two groups (18). 

AL-Inany *et al* in a systemic review assessed the safety and efficacy of subcutaneous rHCG. They evaluated 4 trials including 747 participants, and concluded that there were no statistically significant differences between groups regarding ongoing and delivered pregnancy rate per women (24.1% in the rHCG group and 22.8% in the uHCG group). 

They reported the rate of severe OHSS 3.3% in the rHCG group versus 1.9% in uHCG group (19), similar to this finding ,we showed that risk of OHSS was the same in two group. Our data demonstrated that injection of 250µg of rHCG (Ovidrel) is equal to an intramuscular injection of 10000 IU of uHCG in inducing final follicular maturation in ICSI cycles. We would, therefore, recommend that people under treatment for problems created by opium during the withdrawal process receive special attention to testicular atrophy through consultation with an urologist alongside treatment for other mental and physical side effects.

## Footnote

***Registretion ID in IRCT: IRCT201107034339N2***
